# A thematic analysis of UK COVID-19 vaccine hesitancy discussions on Twitter

**DOI:** 10.1186/s12889-024-21125-0

**Published:** 2025-01-07

**Authors:** Reeshma Jameel, Sheila Greenfield, Anna Lavis

**Affiliations:** 1 College of Medicine and Health, University of Birmingham, Birmingham, UK; 2 Department of Applied Health Sciences, University of Birmingham, Birmingham, UK; 3 Department of Applied Health Sciences and Institute for Mental Health, University of Birmingham, Birmingham, UK

**Keywords:** COVID-19 vaccines, Vaccine hesitancy, Qualitative research, UK, Twitter, Online ethnography, Thematic analysis, Social media

## Abstract

**Background:**

Following UK approval of the Pfizer-BioNTech and Oxford-AstraZeneca vaccines on 2/12/20 and 30/12/20 respectively, discussions about them emerged on the social media platform Twitter, (now ‘X’). Previous research has shown that Twitter/ X is used by the UK public to engage with public health announcements and that social media influences public opinions of vaccines, including COVID-19 vaccines, globally. This study explored discussions on Twitter posted in response to the UK government’s posts introducing the Pfizer-BioNTech and Oxford-AstraZeneca vaccines. The aim was to investigate vaccine hesitant views, and thereby identify barriers and facilitators to COVID-19 vaccine uptake in the UK.

**Methods:**

Online ethnography was used to collect responses (‘tweet replies’) to 14 Twitter posts published by officials or departments of the UK government on the dates the Pfizer-BioNTech and Oxford-AstraZeneca vaccines received approval from the Medicines and Healthcare products Regulatory Agency (2/12/20 and 30/12/20, respectively). 16,508 responses were collected and those expressing vaccine hesitancy were analysed using reflexive thematic analysis.

**Results:**

Three themes that underpinned Twitter posters’ vaccine hesitancy were identified: (1) Concerns about vaccine development and safety, (2) Information, misinformation and disinformation, (3) Distrust: Politics and ‘Big Pharma’. From these themes, eight barriers and eight facilitators to UK COVID-19 vaccine uptake were identified.

**Conclusion:**

This paper highlights key obstacles to COVID-19 vaccine acceptance in the UK based on views from Twitter and contributes to the emerging literature on the relationship between social media and the public response to COVID-19 vaccines. In so doing, this analysis offers insights that are useful for the development of vaccine communication strategies more broadly, both in and beyond future pandemics, to ensure that public concerns are addressed, and misinformation and disinformation are appropriately countered.

**Supplementary Information:**

The online version contains supplementary material available at 10.1186/s12889-024-21125-0.

## Background

The coronavirus (COVID-19) pandemic gave rise to significant global efforts to develop vaccines quickly and effectively [1]. As of November 2024, eight have received approval from the Medicines and Healthcare products Regulatory Agency (MHRA) for emergency use in the UK: the first two, Pfizer-BioNTech and Oxford-AstraZeneca, were approved on the 2nd and 30th of December 2020, respectively, nine months after the pandemic began [2–4]. The UK was the first country to permit emergency use of the Pfizer-BioNTech COVID-19 vaccine [2]. It also approved three bivalent COVID-19 booster vaccines, which target the original COVID-19 strain as well as its subsequent mutations [5–7] A further vaccine rollout for immunocompromised and vulnerable individuals has taken place in Autumn of 2021, and Spring and Autumn of 2022, 2023, and 2024 [6, 8], and certain vaccines are mandatory for health and social care workers, including the COVID-19 vaccine [9]. Hence, while COVID-19 is no longer a global health emergency, its impact is widespread and ongoing [10], requiring continuous vaccine uptake amongst specific designated groups.

By August 2022, greater than 90% of individuals aged 12 years and above had been given a free COVID-19 vaccine [11]. The most popular COVID-19 vaccines in the UK in 2021 were the Pfizer and AstraZeneca vaccines. However, after 2021, booster doses have predominantly been the Pfizer and Moderna vaccines [12]. COVID-19 vaccines were free until August 2023. Since then, free COVID-19 vaccines are only available in the NHS for certain high-risk populations [13]. During the pandemic, some businesses offered incentives for customers who had received a COVID-19 vaccination to support the government vaccination scheme [14]. The vaccine rollout continued through the national lockdown period of January 2021 – July 2021 and beyond [15]. During the beginning of the rollout, a national survey found that 84% of the UK population possessed a positive sentiment regarding COVID-19 vaccination [16]. However, incidents, such as Boris Johnson’s illegal gatherings in 2022 may have weakened the UK population’s trust in the government and may have contributed to vaccine hesitancy in some individuals [17].

COVID-19 vaccine hesitancy was defined by the World Health Organisation (WHO) as deferring or rejecting COVID-19 immunisation despite vaccine accessibility [18]. It is now known that hesitancy towards COVID-19 vaccines was present in the UK during the pandemic [19], with a 2021 study finding that that 31% of UK participants expressed COVID-19 vaccine hesitancy [20]. Vaccine hesitancy more broadly is a key public health issue; in the context of COVID-19, it impeded the efficacy of measures to manage the pandemic [20]. It also impacts public health by decreasing trust in health professionals and governments and, if left unaddressed, can promote suboptimal health literacy [21]. Whilst the uptake of the flu vaccine in England was around 71–75% between 2004 and 2020 and has since remained above the WHO target of 75% [15], in the UK, there has recently been a declining uptake of the Measles, Mumps and Rubella (MMR) vaccine in children, which have resulted in recent measles outbreaks [22]. Between 2011 and 2021, MMR vaccine uptake at 24 months consistently surpassed 90%; however, between 2021 and 2023, uptake declined to approximately 89% and reached the lowest rate since 2009 of 88.9% in 2024 [23, 24]. The current vaccination regime in the UK consists of multiple doses of vaccines given from childhood. There is a comprehensive schedule, but it is not mandated [25].

It is known that social media consumption increased during the COVID-19 pandemic and that social media considerably influenced public opinions of COVID-19 vaccines [19, 26, 27]. This latter aligns with a larger body of evidence regarding the impact of social media on attitudes towards, and uptake of, childhood vaccines and those for other diseases, such as H1N1 influenza [28, 29]. It is known that social media is used to share misinformation around vaccines [26], and it can be challenging for individuals to verify the vaccine-related information they encounter on social media [30, 31]. In tandem, research suggests that people whose primary source of COVID-19 information is social media have a higher likelihood of experiencing vaccine hesitancy [32]. A 2020 multi-country research study found that reductions in the rates of vaccine uptake significantly correlated with foreign misinformation campaigns on social media [27]. Importantly, it has also been suggested that there was, from early in the pandemic, an ‘infodemic’ surrounding COVID-19, with a substantial component of that being vaccine misinformation and disinformation spread through social media platforms [19].

It is key to situate analysis of vaccine hesitancy on Twitter/X, specifically. There are more than 24 million UK-based users of X [33, 34], indicating that it is one of the most popular social media platforms in the UK. Following MHRA’s approval of the Pfizer-BioNTech and Oxford-AstraZeneca COVID-19 vaccines, the UK government used X, then Twitter, as one of the ways to inform the UK public about them. This, then, led to extensive discussions on the platform [26]. To date, a few studies have explored COVID-19 vaccine discussions on Twitter, but they largely have some limitations, including low sample size, exclusion of comments, limited transferability to an UK context and geographic bias [26, 34–43]. For example, Hussain et al. [26]. utilised sentiment analysis to examine US and UK Twitter and Facebook conversations on COVID-19 vaccines during March to November 2020. The study found there to be fewer negative sentiments (22%) in comparison to positive ones (58%), but usage of artificial intelligence (AI) for analysing data may be a limitation, as subtleties and irony cannot be acknowledged, which may have led to some misinterpretation. As such, the study’s authors stated that the impact of confounders, such as political remarks, ought to be taken into consideration and attitudes towards different vaccine companies explored [26]. A multi-country study investigating COVID-19 vaccine hesitancy on Twitter throughout the month following the introduction of the Pfizer-BioNTech vaccine in the UK also used a machine learning approach [30], as did Lyu et al. [36]. A study conducted between July 14 to December 31, 2021, used machine learning-based analysis to investigate 24,063 tweets on the COVID-19 vaccine mandates in the U.S. The study found that a third of individuals on Twitter approved of the mandates while the remainder voiced various concerns regarding the mandates, such as whether there were political agendas involved, the potential violation of human rights and the vaccine’s inability to reduce virus transmission [41]. The study demonstrates that from the initial emergency vaccine approval in December 2020, many concerns of the vaccines found by other US studies persisted, suggesting initial vaccine communication strategies that targeted Twitter users were ineffective. It also highlights the need for effective strategies that directly address users’ concerns. Our study aims to categorise users’ immediate concerns, which may be helpful in increasing the likelihood that future vaccines strategies are more effective.

Furthermore, one multi-national study investigated 664,477 tweets on COVID-19 vaccines from India, UK, Australia and South Africa using sentiment analysis. They found that India had the greatest number of negative sentiments (58.48%) and South Africa had the lowest of 38.88%, while the UK had the greatest number of positive sentiments (21.09%). The study excluded tweets that were not written in English; were less than five words; consisted of only special characters; or had religious or political connotations [39]. Thus, the findings are likely to portray an inaccurate and skewed representation of the perspectives of COVID-19 vaccines on Twitter, as many different languages are spoken in India, verbal communication on Twitter can include different forms and religious and political issues are widely present in the online COVID-19 vaccines discourse. Hence, our study does not include any length, special character or religious restrictions and uses a large sample size.

To date, there have been a few qualitative analyses of COVID-19 vaccine discussions on Twitter. Mwendwa et al. [35]. used thematic analysis to conduct a study on tweets from Africa on COVID-19 vaccines between the 11th and 16th of December 2020. They found themes pertaining to the safety, efficacy and availability of COVID-19 vaccines [35].

A study conducted in Sweden between January to March 2022 found that Swedish-speaking individuals on Twitter generally adopted negative stances towards COVID-19, especially the booster ones [38]. Opinions on COVID-19 vaccines are constantly evolving, hence the findings are only applicable to that 3-month period. A study conducted in Finland analysed 10,724 Facebook comments and tweets from March-May 2021 [40] relating to COVID-19 vaccine uptake. The study may suffer from selection bias due to lack of data available on users’ characteristics available. This means that generalisability is limited, and it is difficult to develop vaccine promoting strategies aimed at specific subgroups of the population using results from the study. It is known that certain issues related to the vaccines are more prevalent in specific age groups and the most effective vaccine communication approaches are those that are tailored to their audience [42]. Our study focuses on the initial impression individuals had towards vaccines, which are likely to be common questions individuals of all ages share, and thus would help inform initial vaccine communication strategies.

A weakness of several of the aforementioned studies was the identification of suitable tweets only by searching for specific key terms and/or hashtags [26, 31, 35–37]. Some studies also excluded tweets that did not have an identifiable geographic origin [26, 31, 34, 35, 40]. As a result, tweets and replies from Twitter users who did not use the key terms, as defined by the study authors, or who did not include their location on their profile were not analysed, possibly introducing selection bias.

A UK study investigated people’s opinions on COVID-19 vaccines in Nottinghamshire by using thematic analysis to analyse 3508 Facebook and Twitter comments posted under ten local organisations during September to October 2023. Themes identified included distrust in the vaccine information available; safety concerns; efficacy concerns; vaccines’ mechanism of action concerns; perceived risk of COVID-19; and immunisation rights. The study suggested that focused communication approaches should be provided by authoritative figures and should target knowledge gaps, address public concerns like adverse effects and highlight the advantages of taking the vaccine. Moreover, they suggest that the approaches should not fuel existing misconceptions and not employ scaremongering to maintain authenticity and gain the public’s trust [43]. This approach would also enable the public to have a more informed perception of the risk of COVID-19. Increased accessibility to vaccines was also suggested as a future recommendation [43].

To date, no UK studies have investigated what Twitter discussions occurred about the introduction of the Pfizer–BioNTech and Oxford–AstraZeneca COVID-19 vaccines in the UK. Lyu et al. [36] along with Fazel et al. [44] found that the volume of tweets about COVID-19 vaccines was greatest following key news announcements regarding COVID-19 vaccines, which illustrates the relevance of conducting research at these time periods. Hence, to address the current gap in the literature, our study analysed 16,508 responses (‘tweet replies’) to public Twitter posts written by officials or departments of the UK government announcing the approval of the Pfizer–BioNTech and Oxford–AstraZeneca vaccines. With a specific focus on investigating vaccine hesitant viewpoints, we aimed to analyse the UK population’s early reactions to the roll-out of these vaccines and thereby gain insights into the facilitators and barriers to their uptake. As an individual’s initial impression may significantly sway their attitude towards future COVID-19 vaccines [20, 21], these tweets offer insights that remain relevant, as COVID-19 vaccine rollouts for specific populations are still ongoing [6]. Moreover, through this analysis, we seek more widely to inform the relationship between Twitter discussions and vaccine hesitancy, offering insights relevant to future immunisation programmes both for COVID-19 and more broadly.

## Methods

### Study design

This paper reports findings from an ethnographic study exploring UK-based online discussions of the pandemic, from March 2020 to October 2022. As part of the study, targeted online ethnographic data collection was undertaken when the UK government announced various COVID-19 regulations and guidelines, such as those around face coverings, lockdowns, and vaccines [45]. As the aims of this study were exploratory, data collection and analysis were qualitative [46], and the study conforms to the Standards for Reporting Qualitative Research (SRQR) guidelines [47].

Grounded in the interpretive framework of medical anthropology, which explores the cultural and social contexts to health and healthcare [45, 48, 49], online ethnography is a well-established method that has been utilised by previous studies to analyse tweets, and social media interactions more broadly [45, 50]. Taking an ethnographic approach meant that the focuses of study were emergent and iterative, defined by societal and political events around COVID-19 as they occurred in the UK in real-time, such as the introduction of lockdowns and the mandating of mask wearing. Ethnography allowed us to “follow” [51] discussions on specific topics across platforms, taking account of interactions, nuances and contexts [45]. During data collection comments were therefore not removed from the original post, with threads of conversations collected. In addition, forms of expression - such as emojis and images- were also collected [45].

### Setting

X, then Twitter, was selected for this study in preference to other social media platforms because of its unique characteristics: it is a microblogging website, where users can publish and share posts of up to 280 characters in length, so many viewpoints can be collected; the platform is designed to promote dialogue between individuals about shared interests; it is able to instantly display a cross-section of reactions to the latest news, including announcements about public health [42, 52]; it is a website used officially by the UK government to disseminate public health information, and was used in this way throughout the pandemic [53]; analysing posts (‘tweets’) offers insights into, and identification of, patterns in the attitudes about the COVID-19 pandemic of different subgroups of the population [54].

### Data collection

Fourteen Twitter posts regarding the approval of COVID-19 vaccines published by officials or departments of the UK government on the days when the Pfizer–BioNTech and Oxford–AstraZeneca COVID-19 vaccines received approval from MHRA (2/12/20 and 30/12/20, respectively) were collected [2, 3]. Key figures were chosen based on their significance in the UK government, and government departments were selected based on their relevance to public health. Other criteria for choosing Twitter posts were also applied. These were: if the original post had sufficient engagement, which refers to the number of replies, ‘likes’ (heart icons), and ‘retweets’ (reposts) a Twitter post receives; included different communication formats, like video recordings with supplementary captions; or if they were the sole posts published by relevant officials or departments of the UK government on those days. The chosen Twitter posts along with their publication dates and number of replies are shown in Table 1. Individuals articulated their views about COVID-19 vaccines under these posts; therefore, as this study aimed to ensure the dataset was rich and contained a variety of viewpoints, responses were collected. Exclusion and inclusion criteria for collecting tweet replies (responses) (Table 2) were applied. Primarily political or spam responses, or those that were posted under any of the 14 chosen Twitter posts later than two months after the 14 initial tweets (Twitter posts) had been published, were excluded from data collection as such responses did not accurately represent the COVID-19 vaccine discussions that occurred during Winter 2020. Automated responses were not excluded because it is impossible to identify them with perfect precision, and individuals could have engaged with them; this means such responses would still be part of the COVID-19 vaccine dialogue [55]. Thus, 16,508 responses were collected after inclusion and exclusion criteria were applied.

**Table 1:** Details of the 14 chosen Twitter threads, including their date of publication and the number of replies they contain

Author	Number of comments
**2nd December 2020 (Pfizer-BioNTech COVID-19 vaccine approved in UK)**	
Matt Hancock	5754
Boris Johnson	1796
Boris Johnson	904
Professor Chris Whitty	497
Department of Health and Social Care	324
Department of Health and Social Care	236
Sir Patrick Vallance	36
**30th December 2020 (Oxford–AstraZeneca COVID-19 vaccine approved in UK)**	
Boris Johnson	3284
Matt Hancock	2417
Matt Hancock	978
Department of Health and Social Care	888
Sir Patrick Vallance	439
Professor Chris Whitty	343
Matt Hancock	173

**Table 2:** Exclusion and inclusion criteria for analysis of responses (‘tweet replies’). A ‘Twitter thread’ refers to a Twitter post and its associated replies. ‘Political’ is defined as any reference made to ‘politics, governments, or public affairs’, in accordance with the Oxford Dictionary [56]

Study Inclusion Criteria	Study Exclusion Criteria
The tweet reply was posted in any of the 14 chosen Twitter threads written by officials or departments of the UK government on 2/12/20 or 30/12/20.	The tweet reply was not posted in any of the 14 chosen Twitter threads written by officials or departments of the UK government on 2/12/20 or 30/12/20.
The tweet reply is written in the English language.	The tweet reply is not written in the English language.
The tweet reply is about COVID-19 vaccines.	The tweet reply is not about COVID-19 vaccines.
The tweet reply was posted in any of the 14 chosen Twitter threads within two months of the thread appearing.	The tweet reply was not posted in any of the 14 chosen Twitter threads within two months of the thread appearing.
The tweet reply is not exclusively political.	The tweet reply is exclusively political.

### Analysis

Taking the responses relating to both Pfizer–BioNTech and Oxford–AstraZeneca COVID-19 vaccines as a single dataset, the whole dataset was then read and searched for vaccine hesitant responses, which were those which aligned with the WHO’s definition of COVID-19 vaccine hesitancy: “the delay in acceptance or refusal of vaccination despite availability of vaccination services” [18]. All the tweets that directly expressed, had connotations of, or were in response to other tweets that expressed COVID-19 vaccine hesitant views were then included in the analysis.

Reflexive thematic analysis was employed due to its flexibility as it allowed textual and visual content to be part of the analysis [57]. This is crucial to the data collected through online ethnography, which includes all forms of expression on social media. Appendix 1 contains Table S1 which illustrates the generated codebook. Appendix 2 contains a visual map displaying how initial subthemes were reviewed and final themes were formed.

Once the themes had been generated, an additional step was undertaken, to identify barriers and facilitators to the uptake of COVID-19 vaccines in the UK. All themes and subthemes were analysed for barriers and facilitators.

The principles of transferability, credibility, and reflexivity were addressed to demonstrate trustworthiness in this study, following guidance on best practice by Korstjens [58]. Transferability was ensured through extensive description of the study setting and methods [58, 59].

To enhance credibility throughout data collection and analysis, the first author made ethnographic fieldnotes about their observations and developing analysis of the data, following guidance by Emerson et al. [60]. Continual engagement with the emerging literature on COVID-19 vaccines facilitated contextualisation and comprehension of tweet replies. The first author also revised codes, subthemes, and themes during analysis [58, 59, 61].

In addition, investigator triangulation was employed to ensure the credibility and validity of the study’s findings. Two Twitter threads were independently coded by the first, second and third authors and discussions were held to review the emerging findings. Results were compared and discussed until agreement was achieved, thereby following reflexive practice to reduce the possibility of bias being incorporated during analysis [58, 59, 61].

To additionally reduce researcher bias, reflexive journals containing thoughts, feelings and assumptions were maintained throughout this study, both during data collection and data analysis; this permitted the research group to consider in a critical manner how their perspectives might have affected the analytical process [58, 59, 62, 63].

### Ethics

#### Consent and anonymisation

Ethical practice is a key consideration when investigating online discourse, as there is an ongoing dialogue about what is classified as public or private data; a core consensus is that this classification is determined by the degree of privacy an individual user expects from a social media platform. It is unreasonable to assume that users of Twitter would acknowledge that their tweets may be examined for research purposes [64]. According to the British Psychological Society (BPS), there is a minimum level of traceability and privacy that social media users can expect; therefore, studies that analyse data from social media platforms must take this into consideration and act accordingly to respect individuals’ expectations of privacy [64, 65]. Nevertheless, the BPS states that observation of online spaces without having obtained prior permission is acceptable when “it is reasonable to argue that there is likely no perception and/or expectation of privacy” [65], meaning analysis may be conducted on public Twitter posts if it abides by the platform’s privacy policy. Hence, in this study, public tweets were considered to be those accessible without logging in, and only tweets responding to the 14 original posts were analysed. As best practice was followed, formal permission from Twitter users involved in this study was not required. Obtaining such consent would have been impracticable and possibly intrusive: it may have resulted in needless distress and individuals being cautious about interacting with future public health posts.

Moreover, to maintain the anonymity of individuals engaging in these Twitter threads, the results section of this paper contains direct quotations only where these are expressions frequently stated by multiple individuals on the platform and so are not traceable to any specific user. In addition, paraphrasing is used to convey the meaning of users’ statements without rendering them traceable. This method for protecting participants that has been employed in other online ethnography studies [64]. It means that some tweet replies have been rephrased by using synonyms and making minimal alterations to the original sentence structure before being presented as quotes in the results section. In addition, no usernames have been included in this paper.

The University of Birmingham STEM ethics committee granted ethical approval for this study in March 2020 (ERN_20–0425).

## Results

Three themes along with their associated subthemes resulted from data analysis:

1. Concerns about vaccine development and safety.
2. Information, misinformation and disinformation.
3. Distrust: Politics and ‘Big Pharma’.

### Concerns about vaccine development and safety

#### Development concerns

Across the dataset, twitter users rejected COVID-19 vaccines with the words, “no thanks,” often following this by citing various reasons. A frequent reason given was concern about both the rapidity of the vaccines’ development, and their effectiveness. Users stated that they felt that the vaccine trials were “incomplete”, and so the vaccines were “experimental”, “rushed”, and had not been “thoroughly tested”.

Ask yourself what shortcuts were taken when normally vaccines take at least 10 years to make. – Reply to Pfizer-BioNTech post.

Such comments were often accompanied by images of COVID-19 vaccine trial data that were misrepresented, such as statistics taken out of context. Users also shared an image of an online article stating that the Oxford-AstraZeneca COVID-19 vaccine trial had an expected study completion date of “2023” [66] and questioned what this meant.

Some users requested “more credible information”:

Can you [government health department] disclose the methodology for the trials first, so I can give informed consent? – Reply to Pfizer-BioNTech post.

Questions were also raised about why treatments for other diseases which had existed for longer than COVID-19, such as “cancer” and “influenza”, had not yet been developed:

10 months for this vaccine, yet half a century of cancer research and still no cure? – Reply to Pfizer-BioNTech post.

Some users asked how it was possible to “isolate” COVID-19 to develop vaccines:

Didn’t the cdc [Centers for Disease Control and Prevention] say they were having difficulty isolating the virus? Yet somehow you’re injecting it into people. – Reply to Pfizer-BioNTech post.

Others questioned what information was available to MHRA that enabled them to approve the Pfizer-BioNTech vaccine before other regulators:

How was the UK able to authorise the vaccine so quickly compared to other countries? What does MHRA know that other regulators don’t? – Reply to Pfizer-BioNTech post.

Others were dubious about MHRA’s approval of the Pfizer-BioNTech vaccine because of a news article stating, “Swiss regulators” had not approved the vaccine due to “incomplete trial data”. (104)

A few commented that the COVID-19 vaccines had been “fast-tracked” due to “Brexit”, meaning that the “EU’s regulations for vaccine approval” did not apply to the UK.

In addition to concerns around the development of the vaccines, twitter users expressed concerns about their “efficacy.”

How much protection does a single dose provide? – Reply to Oxford-AstraZeneca post.

A few users also asked whether the COVID-19 vaccines would still be “effective” against COVID-19 variants.

Part of these discussions of efficacy centred on perceived “differences” between the Pfizer–BioNTech and Oxford–AstraZeneca vaccines. Users remarked that the Oxford–AstraZeneca vaccine was “substandard” to the Pfizer-BioNTech one due to its lower “cost” and “62% efficacy”. Some therefore doubted whether the UK COVID-19 vaccination plan was influenced more by scientific evidence or economic factors:

Did you [politician] decide which vaccine to roll out based on cost over effectiveness? – Reply to Oxford-AstraZeneca post.

Evidencing specific vaccine hesitancy, rather than anti-vax attitudes, this uncertainty around efficacy led some users to want to “know” and “choose” which COVID-19 vaccine they would receive.

#### Safety concerns

Against the background of uncertainty amongst Twitter users related to the vaccines’ development, safety concerns were noted by many users expressing COVID-19 vaccine hesitancy. References to the UK population acting as “guinea pigs” for COVID-19 vaccines were made frequently and “deaths” or severe “adverse events” that had occurred in the COVID-19 vaccine trials were mentioned as reasons to doubt the vaccines.

The safety standards were lowered. They [MHRA] anticipate many adverse reactions. – Reply to Pfizer-BioNTech post.

In addition, users asked whether the vaccines were “safe for pregnant women” and individuals with an “autoimmune disease”. Various tweets expressed concern about the contents of the vaccines, especially with regards to “allergies” and “animal products”.

A frequent component of safety concerns was uncertainty regarding the short-term and “unknown long-term side effects” of the vaccines. Again, illustrating vaccine hesitancy rather than necessarily, more entrenched anti-vax attitudes, some users stated that once the vaccines’ long-term safety had been determined, they would consider taking them:

I will skip it for now and see later after we know more about side effects. – Reply to Pfizer-BioNTech post.

Some Twitter users expressed concerns about “reports of MHRA” needing an “AI system” to record “large numbers of side effects.” Of the latter, the vaccines’ potential longer-term effects on “fertility” were frequently highlighted and discussed:

Beware, it is unknown whether the COVID vaccine impacts fertility!! – Reply to Pfizer-BioNTech post.

Some users compared the potential long-term consequences to those of “asbestos”, “thalidomide” and other theories that were shared by users included beliefs that COVID-19 vaccines would “modify” people’s “DNA”, contained “microchips”, or would be used for “depopulation” or “population control” purposes by rendering those who take it “infertile” in the long-term. In addition, some users were concerned about the vaccine potentially causing “autism”, “decreased motor skills”, “paralysis”, “death” and “body deformations”.

Notably, the novelty of a “mRNA” vaccine was highlighted by some, who urged others to “wait” because they claimed the long-term safety and side-effects of such vaccines had not yet been “studied”.

### Information, misinformation and disinformation

#### Uncertainty

In addition to the uncertainty over the vaccines’ development, safety and side-effects, Twitter users discussed their vaccine hesitancy as arising from uncertainty around many other aspects of the vaccines. Often requesting clarification from others on Twitter, users asked for example, who cannot have COVID-19 vaccines, how many doses would be required, the vaccines’ impact on COVID-19 restrictions, the potential enforcement of “mandatory” vaccination and any “repercussions” for remaining “unvaccinated”.

There was widespread uncertainty around the mechanism of action of COVID-19 vaccines, such as whether they prevented “viral transmission”. This resulted in some users justifying not wishing to receive the vaccine by stating that, since vaccines do not “stop the spread” of COVID-19, there was “no point” in taking them. Others responded to these comments, explaining that COVID-19 vaccines would “reduce the symptoms” of COVID-19.

Against this background, some users suggested that more “education” about COVID-19 vaccines by “health professionals” would be useful. One commented that an explanation from a doctor about how the vaccines work had improved their understanding:

My doctor pal explained that it doesn’t prevent you from getting COVID but reduces symptoms. So rather than your lungs failing, you will have a sore throat & mild cough. – Reply to Oxford-AstraZeneca post.

This is illustrative of a wider trope amongst twitter users; many stressed the importance of “doing your own research” to make an informed decision about the COVID-19 vaccines. They discussed their findings and shared links with others in order to dissuade or persuade them about vaccination. Users often supported these discussions by referencing their sources of information or quoting statistics.

Some negative attitudes towards COVID-19 vaccines appeared to be influenced by vaccine hesitant opinions expressed by public figures, such as “Mike Yeadon”, who is a semi-retired British anti-vaccination activist that worked at the pharmaceutical company, Pfizer [67]:

Have a look at the facts by Dr Mike Yeadon before you become so confident about the vaccine. – Reply to Pfizer-BioNTech post.

#### Questioning the need for the vaccine

In Twitter discussions, some users questioned the need for the vaccines at all, arguing that COVID-19 only seriously affected the “elderly”, and therefore questioning why younger individuals needed them. Many also concluded the vaccines to be “unnecessary” because of COVID-19’s “high survival rate”:

We don’t need help, 99.7% will be fine with mild/no symptoms. – Reply to Pfizer-BioNTech post.

In these discussions, users expressed the view that COVID-19 was not a serious disease, with many stating that they “trusted” their “immune system” to overcome it and perceived it to be “better” and “safer” than taking vaccines.

Others expressed the belief that COVID-19 will never be eradicated and will be similar to the influenza virus and the common cold so there is “no point” in taking a “rushed” vaccine when re-infection is not guaranteed.

In addition, many tweet replies expressed dislike for what they saw as paternalistic COVID-19 policies and vaccination “coercion”, which were described by some as “unethical”:

We demand back our HUMAN RIGHTS. – Reply to Pfizer-BioNTech post.

Healthy individuals forced to take unnecessary vaccines…crimes against humanity. – Reply to Pfizer-BioNTech post.

#### Distrust: politics and ‘big pharma’

#### Distrust in COVID-19 vaccine manufacturers

Linking back to the uncertainty around the vaccine, noted above, some social media users distrusted COVID-19 vaccine manufacturers, like Pfizer. They believed that these vaccine manufacturers should be “liable” for any serious vaccine-induced “adverse reactions” and questioned “why” the UK government had “granted indemnity” to these vaccine manufacturers if the vaccines were “completely safe”. Reference to Pfizer’s past pharmaceutical settlements were made as a reason to distrust them:

“#Pfizer agreed to pay $2.3 billion criminal fine. See below [link to reference article].” – Reply to Pfizer-BioNTech post.

Reference to various theories were made by users. This included the “Big Pharma” theory, in which “pharmaceutical corporations” were thought to prioritise profitability and act against the public’s best interest.:

Thalidomide was approved to be safe, but what does a couple of limb deformities matter to these greedy big pharmas? - Reply to Pfizer-BioNTech post.

Users expressed the belief that COVID-19 was a “hoax” and a huge “big pharma operation” used to increase vaccine sales.

#### Distrust in the UK government and health experts

Many users stated that distrust in the UK government contributed to their perceptions of COVID-19 vaccines, specifically in terms of hesitancy.

Several tweet replies cited the government’s prior management of the COVID-19 pandemic as a key reason for distrusting their vaccination plans. Many believed the government was “incompetent”, made “wrong” decisions and implemented “ineffective” COVID-19 policies:

Very hard to believe you [politician] when you’ve previously said that COVID is like a mild flu, masks weren’t necessary and schools were safe. All proven wrong. – Reply to Pfizer-BioNTech post.

There were remarks that the government were telling “lies” about COVID-19 vaccines, linking this to government “accountability” for excess COVID-19 related “deaths”:

Stop with all the lying…70,000 + have died because of you [politician]. – Reply to Pfizer-BioNTech post.

Users criticised what they saw as they believed that there was excessive COVID-19 “scaremongering” on the part of governments and medical authorities:

“We won’t listen to your [government officials’] scare statistics.” – Reply to Pfizer-BioNTech post.

Some users believed that the UK COVID-19 vaccination plan was influenced by politics rather than evidence. Some were concerned about vaccine politicisation and felt the “timing” of the Pfizer-BioNTech vaccine approval announcement was suspicious, suggesting the announcement was a “distraction” to “cover up” issues related to the UK’s management of the pandemic:

Coincidental timing for news on vaccines, right when tiers start. – Reply to Pfizer-BioNTech post.

Many believed that that there were ulterior political motives behind the rapid mass vaccination of the UK public. Some claimed that politicians had vested interests behind the COVID-19 vaccine announcements; these users claimed that COVID-19 vaccine-related “contracts” would be given to “friends” of politicians, citing news reports of previous instances where this had occurred as evidence. Concerns were raised about government officials potentially having “conflicts of interest” regarding vaccine promotion for instance, by owning shares in COVID-19 vaccine companies:

I suppose your [government adviser’s] share prices are doing good? – Reply to Oxford-AstraZeneca post.

Many users therefore criticised UK politicians’ tweets and speeches advocating COVID-19 vaccines. Some demanded that politicians should “lead by example” by being the first cohort to receive the vaccines:

You [politician] and your family take the vaccine first, then I’ll believe you. – Reply to Pfizer-BioNTech post.

Others alleged that even if politicians were vaccinated “live on TV”, they would deceive the public by receiving “placebo” COVID-19 vaccines.

Some users’ distrust in government was linked to broader political scepticism, with references made to the “The Great Reset’ conspiracy theory, suggesting the COVID-19 pandemic was a “hoax” to collapse the global “economy” and introduce “socialist” policies, such as compulsory vaccination.

The language used in the Twitter posts published by UK government officials was criticised by some for being somewhat dispiriting:

You [government adviser] don’t sound too pleased? Wasn’t the vaccine supposed to end this mess? – Reply to Pfizer-BioNTech post.

Interestingly, the vaccine announcement Twitter posts published by politicians received mostly negative responses, while those by individuals with a scientific or medical background received more positive ones.

Positive responses consisted of congratulatory phrases and expressions of gratitude towards those involved in the development of COVID-19 vaccines:

“Thank you, Chris Whitty, and others for everything you’ve done to help make this happen.” – Reply to Pfizer-BioNTech post.

Yet, a minority of users also questioned the trustworthiness of the Strategic Advisory Group of Experts on Immunization (SAGE):

Why would anyone listen to the never-ending lies that SAGE spouts? – Reply to Oxford-AstraZeneca post.

In addition, some users claimed “MHRA were not independent”, citing reports of the “Bill and Melinda Gates Foundation” supplying MHRA with “funding” to enhance the safety monitoring of new treatments in developing nations.

### Barriers and facilitators

From the identified themes and subthemes, 8 factors affecting COVID-19 vaccine uptake were developed, which were further classified into 8 barriers and 8 facilitators to vaccine uptake; each factor was associated with a barrier and facilitator. The factors are concerns about the safety, development, and effectiveness of COVID-19 vaccines; COVID-19 vaccine roll-out; trust levels in the UK government; trust levels in COVID-19 vaccine manufacturers; availability of COVID-19 vaccine information; public attitudes regarding COVID-19 vaccines; perceived necessity of COVID-19 vaccines; and risk perception of COVID-19.

## Discussion

### Key findings and implications

This is the first UK study to provide an insight into the Twitter discussions that occurred in response to the introduction of COVID-19 vaccines in the UK. It offers key insights into the vaccine hesitancy now known to have been present among the UK population. A variety of perspectives were expressed by Twitter users on the Pfizer-BioNTech and Oxford–AstraZeneca COVID-19 vaccines, including vaccine-hesitant, neutral and pro-vaccination viewpoints and this paper has presented the findings related to vaccine hesitancy. Ranging from uncertainty related to the material properties, safety, and efficacy of the vaccines themselves; to statements regarding their lack of necessity due to cynicism about COVID-19; to mistrust of government, experts and big-Pharma, the identified themes and subthemes were closely interrelated and, often, mutually productive. However, the analysis indicated clear barriers and facilitators to COVID-19 vaccine uptake in the UK at the time the first two vaccines were introduced. These findings are of relevance to continued rollouts of COVID-19 vaccines and to understanding vaccine hesitancy more broadly.

Vaccine safety and development concerns were prevalent and were defined in this study as statements that queried or expressed concerns about or gave reasons to doubt the safety or development of COVID-19 vaccines. Twitter users’ concerns about the development, safety and UK distribution of COVID-19 vaccines were widespread and repeated across the two-month timeframe of collected data, but they varied according to the vaccine brand. Users were mainly concerned about the Pfizer-BioNTech vaccine’s novelty and the Oxford–AstraZeneca vaccine’s efficacy. Many questioned the Pfizer-BioNTech vaccine’s rapid speed of development and suggested that it had not been rigorously tested, so could not possibly be safe. Several users also questioned the Oxford–AstraZeneca vaccine’s efficacy and suggested that it was inferior to the Pfizer-BioNTech vaccine and would only be used because of its lower cost.

Some concerns were common to both vaccines. For instance, fears about the long-term side effects of COVID-19 vaccines and the logistics of UK COVID-19 mass vaccination were prevalent for both vaccines. Concerns about COVID-19 vaccines’ effects on fertility were also widespread. This may partially be because initially when the vaccines were approved in the UK, they were not recommended for pregnant women, which was the case at the time of the collected discussions [68].

Safety and efficacy concerns were also reported by Tang et al. [69], who analysed 3,731 Twitter and Facebook comments posted under COVID-19 vaccine-related news articles by six Canadian news companies between July and September 2020 [69]. This suggests that concerns regarding COVID-19 vaccines were consistent across countries.

Hence, targeted as well as global strategies by policy makers to counteract misinformation and vaccine hesitancy is required. Such approaches should be guided by the developing conversations that arise on social media, as this constitutes the platform on which misinformation spreads the fastest [43]. Analysing and monitoring Twitter conversations would offer beneficial insights into the current issues relating to COVID-19 vaccines and our study demonstrates this.

Nevertheless, in our study, the prominence of efficacy rather than side effect concerns regarding the Oxford–AstraZeneca vaccine suggests that in the time between the approvals of Pfizer-BioNTech and Oxford–AstraZeneca COVID-19 vaccines, users had become more accepting of COVID-19 vaccines, as there were fewer discussions about rejecting the vaccines and more consideration of which vaccine to take. It also suggests that attitudes towards COVID-19 vaccination are not fixed. Policymakers should actively identify prevalent public concerns about vaccines, especially as they appear on social media, and promptly address them to positively influence vaccination attitudes.

As COVID-19 vaccination is an emotive subject [70], vaccination programmes could consider using communication strategies that establish emotional connections with the public to be more engaging rather than those that could be considered as ‘fearmongering’. For instance, sharing vivid accounts from those severely affected by COVID-19 about their suffering could elicit emotional responses [66].

Our study also found that attitudes towards vaccine announcements varied according to the announcement tweet’s author: tweets published by politicians received mostly negative replies, while those by current or previous government scientific advisers received more positive ones. These findings align with Lyu et al. [36].

In our study, distrust in both the UK government and COVID-19 vaccine manufacturers influenced many users’ perspectives of COVID-19 vaccines. Based on their opinions about how the government had managed the pandemic and wider perceptions of politicians, some users felt that they could not trust the UK government. This led to a questioning of politicians’ motives for promoting vaccines and whether the government would use COVID-19 vaccines as a political tool to mitigate perceived pandemic management mistakes already made. Expressions of distrust in COVID-19 vaccine manufacturers predominately related to concerns they would not be held liable if people were to experience severe vaccine-induced side effects.

The emergence of distrust and politics as a key theme could be anticipated due to the fact that the dataset of 14 tweets included those from politicians; Twitter is known to be widely used by individuals to interact with and express their opinions about political figures [67, 71]; and throughout the COVID-19 pandemic, the UK government were heavily criticised on Twitter for their pandemic management decisions [72]. However, expressions of distrust were noted in response to Twitter threads by non-politicians as well. This suggests that distrust in the government does not exclusively refer to politicians, which is concerning as trust in the UK’s public health departments and scientific advisers is needed to instil public confidence in the vaccines and increase compliance with health policies [73].

Lack of trust in pharmaceutical companies and their relationship to the government can lead to vaccine hesitancy, with many individuals expressing fear and cynicism about the rapid development of COVID-19 vaccines, which at the time was a novel feat. Many Twitter users believed that the vaccines could not have been thoroughly vetted for safety and efficacy in such a short period and that the companies and public figures who promoted the vaccines must instead be motivated by financial interest. This concern was exacerbated by claims that many politicians hold shares in, or have affiliations with, vaccine manufacturers [74].

The above findings align with Lanyi et al. [75], who used AI to analyse 91,473 tweets posted between 30 November 2020 and 15 August 2021 from users in London to investigate COVID-19 vaccine hesitancy [75], which suggests that distrust in the government and pharmaceutical organisations, as well as safety concerns, persisted months after the first two COVID-19 vaccines had been approved in the UK. This highlights that vaccine communication strategies may have been ineffective in addressing users’ concerns during that early period; it also demonstrates that our study can be used as a baseline to investigate how UK Twitter discussions on COVID-19 vaccines develop over time.

The UK government has attempted to address COVID-19 vaccine hesitancy by, for example, introducing educational initiatives [76]. However, a UK survey conducted between the 7th and 16th September 2021 on a representative sample of 2,482 adults aged 18 years and over who had previously reported being COVID-19 vaccine-hesitant found that 55% stayed unvaccinated [77]. This suggests that while COVID-19 vaccine acceptance in the UK has increased over time, vaccine hesitancy in some individuals persists and they may require targeted interventions, or potentially the development of new interventions, which could focus on social media. As many users in our study criticised the UK government’s decisions about COVID-19 vaccines, trust in the government needs to be increased to help improve vaccine compliance; clearer explanations of the government’s COVID-19 pandemic management decisions, including how the decisions were rooted in scientific evidence, could arguably help achieve this.

Topics discussed by users in our study closely reflected COVID-19 vaccine-related news topics in December 2020. The COVID-19 vaccine dialogue on Twitter rapidly evolved in response to the latest COVID-19 vaccine news [38]. Also, there were discussions about accurate and inaccurate vaccine information. Many posted a variety of queries about COVID-19 vaccines, and discussed their understanding of the vaccines, which was often based on their personal ‘research’. Users discussed the findings of this personal research into COVID-19 vaccines, but in so doing, shared vaccine misinformation and disinformation.

Vaccine misinformation is defined by the CDC as inaccurate vaccine-related information spread by individuals without or regardless of the intention to mislead people, while vaccine disinformation is defined as the deliberate creation and dissemination of false vaccine-related information for malicious purposes [78]. As data about users’ accounts, such as their prior tweets, were not collected to comply with Twitter’s privacy policy, it was not possible to determine the intentions behind users’ tweets. So, false information about vaccines in tweets were deemed vaccine misinformation in this study. However, given the prevalence of vaccine disinformation in the COVID-19 infodemic [19], it is likely to have been present in some tweets. It was also unknown whether tweets containing misinformation or disinformation were posted by ‘bots’ (robots). Previous research suggests that COVID-19 misinformation was disseminated as frequently as accurate COVID-19 information on Twitter [79], but received more engagement from Twitter users [80]. This may explain the prevalence of vaccine misinformation in users’ tweets in this study.

Twitter initially attempted to tackle the spread of COVID-19 vaccine misinformation by identifying and labelling tweets containing misinformation to make others aware, hiding those tweets, and suspending accounts that persistently spread misinformation. Nonetheless, these restrictions were relaxed under the ownership changes in 2022, which could have potential detrimental effects on vaccine literacy due to the abundance and prominence of misinformation [81].

The above findings align with Nuzhath et al. [82], who analysed 1,286,659 public tweets posted between 19 July 2020 and 19 August 2020 and found themes related to all themes in this study [82]. Similar vaccine misinformation and disinformation was found in their study, which suggests that theories about vaccines develop before and remain constant after vaccines become available. However, unlike Nuzhath et al. [82], our study has transferability to a UK setting and can give a more nuanced insights into the specific concerns of the UK public, which would allow for more tailored vaccine communication strategies.

It is suggested that personality traits may be associated with strong anti-vaccine attitudes [83]. However, this relationship is complex and requires more qualitative research, such as studies comprising interviews and focus groups. It is important to understand the socio-cultural contexts of anti-vaccine attitudes to better design targeted and appropriate information campaigns. Future research could also explore whether information was purposefully being misinterpreted or not during the COVID-19 pandemic and develop strategies to support people to recognise misinformation, thereby reducing vulnerability to it. Strategies targeting anti-vaccine attitudes should utilise non-condescending tones to increase engagement.

Nevertheless, our study also found that there were many users who did not possess “anti-vax” attitudes, but instead were simply hesitant about taking these specific vaccines, which is a key finding. Individuals who are undecided about vaccination may read these discussions, which often contain negative vaccination attitudes, and be adversely influenced. However, this also presents an opportunity for public health agencies to favourably affect online vaccination discourse by, for instance, posting educational, pro-vaccine content under vaccine-related government tweets; such content should be accessible and easily understandable as despite the abundance of vaccine information available online, many users in this study, for example, failed to understand how vaccines work.

Users also deliberated about the importance of COVID-19 vaccines in the wider context of how risky they perceived the virus itself to be. Many perceived COVID-19 to be low-risk and therefore believed vaccination against it was unnecessary, so future COVID-19 vaccine promotion should place more emphasis on the vaccines’ importance. Those who had a low-risk perception of COVID-19 desired the freedom to make decisions about COVID-19 vaccines without being coerced. However, motivations for vaccination also included desires to achieve normality in everyday life and protect others.

Prada et al. [84]. employed content analysis to investigate the content of tweets related to COVID-19 vaccines and further investigated the relationship between the kind of Twitter user and the posts they were posting as were posting. The study found that amongst tweets that portrayed a negative opinion with regards to vaccines, 13% related to discussions about how the pandemic was being managed, 13% related to the science behind the vaccines; 33% related to discussion about refuting the vaccines; 29% related to discussions about protesting taking vaccines. They found that tweets that displayed a positive stance to vaccines pertained to the findings of research, immunisation information and the practicalities, such as booking appointments and accessing vaccines. Objective tweets were generally related to tweets reading preventing and managing the spread of COVID-19, those from organisations and those about medical and scientific information. In contrast, subjective tweets related to those expressing anti-vaccine views [84]. However, a finding from our study is often misinformation can relate to medical and scientific information that has been misinterpreted.

The study concluded that there is a large proportion of tweets that display vaccine hesitancy, mainly due to due to distrust in the government [84]. The concordance of this theme in our study supports the recommendation that strategies that increase the public’s trust in the government, may initially appear to be the most actionable and implementable choice.

### Barriers & facilitators and implications

Barriers and facilitators to UK COVID-19 vaccine uptake were identified. These were about vaccine product concerns, vaccine rollouts, trust levels in the government and vaccine manufacturers, risk perceptions of COVID-19, the availability of vaccine information, vaccine necessity, and public attitudes to vaccines.

The identified barriers and facilitators are concordant with those reported by Liew et al. [37] and supported by a UK cross-sectional survey conducted between 4 September 2020 and 9 October 2020 on 4884 individuals which investigated the factors affecting COVID-19 vaccine uptake [85]; they reported findings related to barriers 1 (safety concerns), 3 (trust in the government), 4 (trust in vaccine manufacturers), 5 (COVID-19 risk perceptions), 6 (vaccine-related information availability) and 7 (vaccine necessity) in this study as well as a finding that some participants rejected the vaccines because of religious reasons [85], which was not found in this study. Our study goes beyond what has already been reported due to the use of manual thematic analysis. By illustrating the key conversations that were being held on Twitter, it allows for online vaccine communication strategies to anticipate the key arguments presented by those experiencing vaccine hesitancy and hence can allow for responses to be developed and interventions to counteract the spread of misinformation. Acknowledging and responding to criticism will help to create an evidence-based narrative and restore the public’s faith in the UK government.

The results of this study are transferable to the UK population since the variety of views expressed by users mirror the general conversations that occurred in the broader public. A UK survey conducted on a representative sample of 18,112 adults aged 16 years and above between 13 January and 7 February 2021 found that that side effect concerns were a top reason for individuals’ COVID-19 vaccine hesitancy [86], which is consistent with this study. Our study added to this existing literature by highlighting key subthemes relating to side effect concerns, such as fertility and unknown long-term implications.

This study’s findings illustrate key public concerns that emerged in December 2020, when two COVID-19 vaccines had just been approved, which need addressing to promote vaccine uptake. People were expressing these concerns on Twitter, so it is useful for policymakers to look at this medium to gauge public opinions on COVID-19 vaccines; this also suggests that Twitter is a useful medium to disseminate government information and dispel myths and misconceptions about COVID-19 vaccines. Therefore, the findings suggest that social media needs to be utilised effectively as part of the UK COVID-19 vaccination programme to increase public confidence in COVID-19 vaccines. There is a need for health professionals, scientists, the UK government, and public health agencies to actively engage with the COVID-19 vaccine dialogue on Twitter. For instance, this could be achieved through disseminating accurate, accessible information related to COVID-19 vaccines frequently on Twitter, promptly responding to or flagging tweets spreading COVID-19 vaccine misinformation and answering vaccine-related queries. These actions can be beneficial in enabling the UK public to make more informed decisions about vaccination against COVID-19 by improving vaccine literacy, which can help increase vaccine acceptance. The discussed recommendations are supported by prior literature, which has reported that effective health communication can favourably influence vaccine acceptance [69].

The findings may also be applicable to vaccines in general as similar concerns were raised about vaccines for other infections. For instance, the Royal Society for Public Health (RSPH) surveyed the attitudes of 2,000 UK adults in 2018 towards vaccines, such as the MMR and Influenza vaccines, and discovered that side effect concerns were the most popular reason for rejecting vaccines [87]. Some weaknesses of the RSPH study include its small, mostly female sample and under representation of ethnic minorities, meaning the findings may not be representative of the UK population.

However, there are some differences in the vaccine discourse for different vaccines. For instance, a global study analysed 285,417 tweets to investigate what discussions occurred regarding Human papillomavirus (HPV) vaccines during October 2013 to October 2015 and found topics related to concepts underpinning the themes in this study, but 57.16% of tweets were about information and advocation [88]. In contrast, fewer informative and positive tweets compared to misleading and negative ones were noted during analysis in this study. This suggests that analysing tweets can identify differences in discussions that occur about different vaccines and therefore could be used by policymakers to assess public attitudes towards vaccines and refine vaccination programmes.

The novelty of this research in comparison to the existing literature is that it is the first UK study to investigate what Twitter discussions occurred about the introduction of COVID-19 vaccines in the UK, and one that uses an ethnographic approach to do so. Furthermore, some of the study findings reveal specific misconceptions about COVID-19 vaccines as well as the evidence that anti-vaxxers use to back up their points, which provides a deeper and more holistic insight compared to existing literature, where analysis has predominantly been conducted by AI or sentiment analysis, which excludes nuances of comments. The implications this research works towards generating is to provide actionable insight into how online users perceive and engage with vaccine discourse. By examining text and nuances of tweets, anti-vaccination responses can be anticipated, and targeted online interventions can be designed to improve vaccine literacy and target misinformation.

This study analysed users’ views about the introduction of COVID-19 vaccines, but it may also be useful to conduct similar analyses on Twitter in response to vaccine ‘scares’, such as when Japan suspended 1.6 million Moderna COVID-19 vaccine doses due to reports of contamination [89]. Negative news reporting can greatly reduce confidence in vaccines [90]. Collecting insights into users’ initial concerns after such incidences would enable vaccine communication strategies to be more responsive to users’ concerns and need for reassurance.

### Future research

To obtain a more holistic picture of the discussions surrounding the introduction of COVID-19 vaccines in the UK, future research on other social media platforms, such as Facebook and Reddit, is required, which may be preferentially used by different demographics. Such research should not impose any language restrictions during data analysis to capture all potential expressed views; data and methodological triangulation should be used to increase the validity of findings [62]. Conversations on these platforms may differ from those on Twitter, since different individuals interact with social media differently and this can be influenced by demographic factors, such as sex, age and socioeconomic status [91]. The COVID-19 vaccine dialogue will also vary by country [55]. Thus, social media discussions on COVID-19 vaccines in the UK and globally should be monitored throughout the pandemic to determine how public perspectives evolve and the effectiveness of any COVID-19 vaccine educational campaigns.

Cooperation between researchers, social media platforms and governments is required to investigate the demographic characteristics and social networks of those who engage in online COVID-19 vaccine discussions [92]. Such research would enable exploration of how COVID-19 vaccine narratives form on social media and could potentially identify vaccine-hesitant groups for interventions to target [93].

### Strengths and limitations

This is the first UK study to investigate what Twitter discussions occurred about the introduction of COVID-19 vaccines in the UK; it has presented novel insights into the COVID-19 vaccine dialogue in the UK as well as identified factors that may influence the uptake of COVID-19 vaccines. Methodological rigour was addressed via use of reflexive journals, investigator triangulation and persistent observation.

Nevertheless, this study has some limitations. The findings strictly apply to the chosen data collection dates; use of a longer data collection period could alter the findings. However, focusing on people’s immediate responses to the Pfizer–BioNTech and Oxford–AstraZeneca COVID-19 vaccines was useful to address this study’s aim. To preserve the anonymity of users, demographic information was not collated; this is a common limitation to social media research [69]. This may have a slight impact on results because there is a chance that some of the responses were posted by individuals who do not reside in the UK. However, we can reasonably presume that the majority are likely to be from UK citizens because many individuals referred to lived experience in the UK in their tweets.

It was presumed that these users would likely reside in the UK, which would increase the study’s transferability to the UK; however, no geographical data was collected to verify their locations. Only responses under 14 Twitter posts written by five accounts were analysed. Users who gathered under these posts may represent a specific subset of the population, which decreases the transferability of the findings. This study has addressed the issues of foreign interferences on social media platforms, particularly during COVID-19, by focusing on Twitter threads from UK-based accounts, which increases the likelihood responses under the posts are from UK residents as they would be the target audience. Nonetheless, it is not possible to guarantee that none of the tweets were posted by bots/AI or foreigners.

The identified barriers and facilitators to COVID-19 vaccine uptake may not be representative of the views of the wider UK public, since only those who had internet access and engaged with Twitter will have participated in the study. Nevertheless, qualitative research does not intend to be generalisable, but rather aims to explore views to provide an in-depth understanding of a subject with regards to a specific context [58], in this study, the subject was discussions that occurred about the UK introduction of COVID-19 vaccines and the context, conversations on Twitter.

Despite the UK being a multi-cultural society [94], tweet replies not written in English were not analysed, so some views may possibly have been missed; however, this resulted in the exclusion of only a minute number of tweet replies. Automated responses were not excluded, so the findings may have been influenced by some non-human responses [95]. A larger sample size could have identified more themes; however, this is unlikely as this study’s findings concur with those of previous larger studies [35, 38, 69] and data saturation was achieved.

## Conclusion

Through its identification of three themes in the data, this study has clearly identified barriers and facilitators to the uptake of COVID-19 vaccines in the UK. These highlight key issues surrounding the acceptance of COVID-19 vaccines in the UK and demonstrate that analysing tweets is an effective way of identifying public concerns about COVID-19 vaccines. These insights may be useful for the development of effective COVID-19 vaccine communication strategies that address public concerns, increase public confidence in COVID-19 vaccines, and counter vaccine misinformation and disinformation.

## Electronic supplementary material

Below is the link to the electronic supplementary material.

Supplementary Material 1

## Data Availability

Anonymisation of data was utilised in this study to ensure tweet replies were not traceable. Therefore, data is presently unavailable to the public to prevent the identification of participating Twitter users and protect their privacy. Upon reasonable request, the dataset analysed in the study may be made available by the corresponding author.

## Abbreviations

MHRA: Medicines and Healthcare products Regulatory Agency
WHO: World Health Organisation
HPV: Human papillomavirus
