# Supplementary material for: A thematic analysis of UK COVID-19 vaccine hesitancy discussions on Twitter
Source: BMC Public Health. 2025 Jan 7;25:61. doi: 10.1186/s12889-024-21125-0 (PMC11705654; doi:10.1186/s12889-024-21125-0)
Supplement: Supplementary file 1 — Supplementary Material 1 [file 12889_2024_21125_MOESM1_ESM.docx]

Appendix 1: Codebook

| **Table S1 illustrates the codes used, their definitions and initial subthemes.** | | |
| --- | --- | --- |
| **INITIAL SUBTHEME** | **CODE** | **DEFINITION** |
| **Concerns about the COVID-19 vaccine product information** | **Benefit vs risk of COVID-19 vaccine** | Acknowledging there are benefits and risks to the COVID-19 vaccine. |
|  | **UK public as guinea pigs** | Reference to the UK population acting as ‘guinea pigs’ for the COVID-19 vaccine. |
|  | **Vaccine trials concerns** | Expressing concerns about the COVID-19 vaccine trials, e.g., incomplete trials . |
|  | **Vaccine development concerns** | Concerns about how the COVID-19 vaccines were developed. e.g., whether they were thoroughly tested. |
|  | **Vaccine side effect concerns** | Concerns about the COVID-19 vaccines’ short-term and long-term side effects. |
|  | **Vaccine effectiveness** | Reference to the COVID-19 vaccines’ efficacy/effectiveness, including difference between single vs two doses. |
|  | **Vaccine contents** | Reference to the contents of the COVID-19 vaccines. |
|  | **AI reporting system for side effects** | Expressing concerns about the reports of MHRA needing an AI system to record large numbers of side effects. |
|  | **Experimental vaccine** | Believing that the COVID-19 vaccines are ‘experimental’. |
|  | **Vaccine temperature requirement** | Questioning why the Pfizer-BioNTech vaccine has a -70C temperature requirement. |
|  | **COVID-19 variants effects on the vaccines** | Asking about the effects of potential COVID-19 variants on the COVID-19 vaccines’ effectiveness. |
|  | **Virus isolate** | Questioning how the coronavirus was ‘isolated’ |
| **COVID-19 vaccine-related comparisons** | **Still no cure for other conditions like cancer or reliable** | Asking why there are still no cures for some conditions like cancer, when vaccines were developed so quickly. |
|  | **Comparisons to thalidomide** | Comparing thalidomide’s side-effects to COVID-19 vaccines’ potential side-effects. |
|  | **Comparison to flu vaccine** | Asking whether the COVID-19 vaccines will need to be taken annually like the flu vaccine. |
|  | **All medications and vaccines carry side-effect risks** | Highlighting those medications and other vaccines also have risk of side-effects. |
|  | **Comparison to Asbestos** | Reference to COVID-19 vaccines carrying risk of serious long-term side effects in the same way as asbestos. |
|  | **Science moved on since Thalidomide** | Rebuttal to comparisons of COVID-19 vaccines to thalidomide stating that science has improved since the thalidomide incident. |
|  | **People normally willing to take other medications** | Highlighting that people are usually willing to take other medications without much thought. |
|  | **Vaccine differences/brand** | Reference to the COVID-19 vaccine brands or differences between vaccine types, which is better. |
|  | **Vaccine availability compared to other countries** | Asking why other countries haven’t approved the COVID-19 vaccine yet. |
|  | **MHRA efficiency** | Reference to MHRA’s efficiency compared to other regulators as the main reason the UK was the first country to approve COVID-19 vaccines. |
|  | **Reasons for vaccines’ quick speed of development** | Acknowledging/accepting the reasons behind the COVID-19 vaccines’ speed of development compared to that of other vaccines. |
| **COVID-19 vaccine rollout concerns** | **Political science vs actual science regarding vaccination distribution/rollout** | Reference to the distribution of the COVID-19 vaccine being politically motivated/ following government agenda rather than based on trial evidence. e.g., delaying second dose for 12 weeks instead of 3 weeks. |
|  | **Accessibility of vaccination** | Reference to how accessible the COVID-19 vaccines were. |
|  | **Vaccination of healthy people** | Reference to vaccinating healthy people not just clinically vulnerable against COVID-19 as being necessary/unnecessary. |
|  | **Vaccination delivery** | Reference to issues/complaints about COVID-19 vaccine appointments and experiences when getting vaccine. |
|  | **Vaccination logistics and distribution** | Reference to the logistics of vaccinating the UK against COVID-19, e.g., need for swift and effective vaccination response/ how the vaccines will be distributed, including who will be prioritised first, why the 5-day delay. |
|  | **Vaccine availability** | Concern about the availability/supply of the COVID-19 vaccine. |
|  | **Teachers priority** | Stating that teachers should also be considered a high priority group |
|  | **Priority of family members of young clinically vulnerable** | Stating that family members of young clinically vulnerable should also be considered a high priority group. |
|  | **NHS too pressured to deliver vaccine effectively** | Questioning whether the NHS will be able to cope with pressure of delivering the COVID-19 vaccine. |
|  | **24/7 people willing to take dose** | Suggesting that vaccination centres should run 24/7 as people are willing to take their dose any time of the day. |
| **Distrust in authorities** | **Distrust in the government** | Not trusting the government. |
|  | **Concerns about conflict of interest of government officials** | Reference to government officials having conflict of interests, e.g., shares in COVID-19 vaccine manufacturing companies or giving contracts to friends. |
|  | **Politicians should have vaccines first/lead by example** | Believing that politicians should take the COVID-19 vaccine first. |
|  | **Placebo vaccine for politicians** | Believing that politicians would take placebo vaccines rather than actual ones. |
|  | **Government is accountable for COVID-19 deaths** | Blaming the government for COVID-19 deaths, believing they should be held accountable. |
|  | **Language used in speeches dissected** | Comments on the choice of words used in the speech posts. |
|  | **Picture of scientist rather than Boris Johnson** | Stating that a picture of a scientist in a lab would be more reassuring than Boris Johnson in the lab to public about COVID-19 vaccines. |
|  | **Trusting scientists and manufacturer** | Reference to having trust in scientists and COVID-19 vaccine manufacturers. |
| **Distrust in organisations** | **Distrust in vaccine manufacturers** | Not trusting COVID-19 vaccine manufacturers. |
|  | **Transparency of vaccination manufacturers** | Reference to COVID-19 vaccine manufacturers as transparent/not transparent. |
|  | **Liability of vaccine manufacturers** | Reference to COVID-19 vaccine manufacturers needing to be liable/ law changes about indemnity. |
|  | **Sage trustworthiness mixed** | Reference to the trustworthiness of SAGE. |
| **Influence of politics on COVID-19 vaccine attitudes** | **Brexit impact on COVID-19 vaccines** | Reference to the impact of Brexit on COVID-19 vaccines, e.g., vaccine fast-tracked due to Brexit. |
|  | **EU red tape** | Referencing the EU’s strict regulations for vaccine approvals as the reason other European countries haven’t approved the vaccines yet. |
|  | **Vaccine announcement covers up existing problems** | Reference to the COVID-19 vaccine announcement being used as a distraction to other pandemic problems/overhyped/ coincidental timing (e.g., tier system introduced recently) |
| **COVID-19 misinformation on social media** | **COVID-19 vaccine conspiracy theories** | Conspiracy theories regarding the COVID-19 vaccines or COVID-19. E.g., vaccine contains microchip, people turn to zombies, birth/facial defects, great reset. |
|  | **Vaccine misinformation** | Reference to COVID-19 vaccine misinformation, including its effects/ importance e.g. leading to deaths or ignorance |
|  | **Facebook unreliable** | Stating that COVID-19 related information available on Facebook is unreliable. |
|  | **Bill Gates involvement** | Reference to Bill gates’ indirect involvement/potential influence in the COVID-19 vaccines stating that MHRA received funding from him. |
|  | **Big pharma** | Reference to Big Pharma and COVID-19 vaccines. |
| **Conversations between Twitter users** | **Rebuttals to COVID-19 vaccine conspiracy theories** | Responding to COVID-19 vaccine conspiracy theories with a rebuttal. |
|  | **Active dissuasion** | Users actively dissuading those who express desire to receive the COVID-19 vaccines. |
|  | **Significant proportion of the public undecided** | Highlighting that a significant proportion of the public are undecided. |
| **COVID-19 vaccine research and understanding** | **Understanding of vaccine profits** | Believing that COVID-19 vaccines are produced at profit/no profit/cost price. |
|  | **Personal research** | Needing to do personal research to form an opinion about the COVID-19 vaccines. |
|  | **Research interpretation** | Same research evidence interpreted differently by individuals. |
|  | **Sources of vaccine information** | Reference to/links to sources of COVID-19 vaccine information. |
|  | **Need to make an informed decision** | Asking for more credible information about COVID-19 vaccines/need for more assurance to make an informed decision. |
|  | **Vaccine mechanism and associated concerns** | Reference to how COVID-19 vaccines work and causes immunity and whether it stops transmission or just reduces symptoms, and concerns, e.g., DNA modification. |
|  | **Herd immunity** | Reference to COVID-19 vaccines causing herd immunity, including queries and links to sources of relevant information. |
|  | **Justified vaccine quick speed of development** | Acknowledging/accepting the reasons behind the COVID-19 vaccines quick speed of development compared to other vaccines. |
|  | **Statistic out of context** | Reference to individuals focusing on a specific statistic out of context |
|  | **Frustration** | Expressing frustration about COVID-19 vaccine information, e.g., sick of experts |
| **Clarification** | **Reliability of antibody tests** | Reference to antibody/PCR tests and whether they accurately or reliably detect COVID-19 antibodies. |
|  | **Changing or contradicting vaccine advice** | Pointing out changing or contradicting advice about the COVID-19 vaccine, e.g., who can have it. |
|  | **Number of jabs required** | Questioning how many COVID-19 vaccine jabs will be required for an individual. |
|  | **Mandatory vaccination** | Reference to the COVID-19 vaccination being mandatory, including queries about it, showing support or being against it. |
|  | **Vaccination cards and passport** | Reference to the potential introduction of COVID-19 vaccination cards/passport, including queries about it, showing support or being against it. |
|  | **Discrimination by vaccination status** | Asking about whether there will be discrimination of those vaccinated and those unvaccinated against COVID-19. |
|  | **Clarification of who can have vaccines** | Asking for clarification about who can have the COVID-19 vaccine, e.g., people with auto-immune conditions, allergies, pregnant women |
|  | **Excessive vaccine training** | Reference to individuals, such as retired nurses, needing excessive vaccine training (20 hours) to deliver the vaccine. |
|  | **Ivermectin for those with covid** | Asking about the effectiveness of Ivermectin for those with COVID-19. |
| **Importance of COVID-19 vaccine** | **Perceived importance of vaccination** | Believing that the COVID-19 vaccine is important/unimportant. |
|  | **COVID-19 risk to public health** | Perception of COVID-19 risk to public, including high and low risk. |
|  | **COVID-10 risk to oneself** | Perception of COVID-19 risk to oneself, including high and low risk. |
|  | **Importance of people's decisions** | Recognising the importance of others’ decisions in managing COVID-19. |
|  | **Utilitarianism** | Believing that mass COVID-19 vaccination is for the greater good of others. |
|  | **Vaccine ineffective due to the low effectiveness of other pandemic strategies** | Believing that COVID-19 vaccine is not important or is ineffective due to the low effectiveness of other pandemic strategies. |
|  | **Low COVID-19 mortality rate so vaccine unnecessary** | Reference to the mortality rate of COVID-19 being low, suggesting that COVID-19 vaccines are unnecessary. |
|  | **Vaccine purpose if no normality** | Questioning the purpose of COVID-19 vaccines if normality will never be achieved. |
|  | **Trust immune system** | Reference to having trust in one’s immune system to protect against COVID-19 without a vaccine. |
| **COVID-19 vaccine motivators** | **Vaccine means normality** | Reference to the COVID-19 vaccine leading to normality in life. |
|  | **Duty to protect others** | Reference to believing they have a duty to protect others by taking the COVID-19 vaccine. |
|  | **Personal motivators** | Reference to personal motivations for taking COVID-19 vaccines. |
|  | **Do your bit** | Believing that dealing with COVID-19 is a team effort and everyone needs to contribute by taking the COVID-19 vaccine. |
| **Influence of others on vaccine decision-making** | **Influence of social media** | Any reference to social media influencing perceptions of the COVID-19 vaccines. |
|  | **Discussions with others** | Having COVID-19 discussions with others. |
|  | **External pressures** | Pressure to get vaccinated against COVID-19 from others: peer-pressure. |
|  | **Vaccine hesitancy among health workers is discouraging** | Reference to some health workers being hesitant about taking COVID-19 vaccines, which discourages the public. |
|  | **May have vaccine after others** | Reference to possibility of having vaccine after others in order to see long-term side- effects, |
| **Pro-vaxxers vs Anti-vaxxers** | **Public are brainwashed** | Believing that pro-vaxxers are brainwashed by government or anti-vaxxers are brainwashed by internet conspiracy theories. |
|  | **Pro-vaxxers are bots** | Reference to pro-vaxxers being bots. |
|  | **Anti-vaxxers stereotype** | Reference to anti-vaxxer stereotypes:  e.g., Gullible, crazy, unintelligent |
|  | **Critiquing = anti-vaxxer** | Reference to people criticising the COVID-19 vaccine meaning they are automatically against vaccines. |
|  | **Those that have doubts simply shouldn't take it** | Believing those that have doubts about the COVID-19 vaccines, should just not take it and stop criticising the vaccines on Twitter. |
|  | **Mike Yeadon wanting to halt approval** | Reference to Mike Yeadon, previous senior figure at Pfizer allergy and respiratory research division and CEO of Ziarco, stating COVID-19 vaccine approval should be halted. |
|  | **Explaining is futile** | Commenting that explaining the safety of COVID-19 vaccines to anti-vaxxers is futile as they are stubborn/won’t reason. |
|  | **Sheep** | Believing that those that accept the COVID-19 vaccine without questioning it are ‘sheep’ |
|  | **Take my dose** | Stating that others can have an individual’s dose if they don’t want to have it. |
|  | **Different vaccine attitudes based on author of announcement** | Majority positive or negative vaccine responses to tweets from different authors, e.g., positive under CMO tweet but negative under Boris Johnson. |
| **COVID-19 vaccines are a good thing** | **Public’s positive attitude towards vaccination** | Any reference to the public having a majority positive attitude towards the COVID-19 vaccines. |
|  | **Gratitude** | Expressing thanks/gratitude for COVID-19 vaccine development, including directly to senior figure and to researchers involved etc. |
|  | **Vaccine news is surface-level good news** | Reference to the COVID-19 UK vaccine approval as being good news on the surface but not great as many pandemic problems won’t be solved by the vaccine, e.g. COVID-19 vaccines don’t solve current problems or stop the virus spreading immediately. |
|  | **Vaccine is great news** | Reference to the COVID-19 vaccine being great news. |
|  | **Collaborative and global achievement** | Reference to the COVID-19 vaccines’ development being an international accomplishment and a team effort. |
|  | **Scientific breakthrough** | Reference to COVID-19 vaccine quick development as being a great achievement for science. |
|  | **Congratulations** | Congratulating those involved in developing/allowing the vaccine to happen. |
|  | **Finally good news** | Reference to the COVID-19 vaccine approval as finally being some good news as most news about COVID-19 is negative. |
|  | **Rest of the world to follow** | Hoping that other countries will approve the COVID-19 vaccines soon. |
|  | **Patriotism** | Being proud about the Oxford vaccine being developed in Britain/UK being the first country to approve COVID-19 vaccines. |
|  | **Need for pubs to reopen** | Expressing desire for pubs to reopen now that vaccines are approved. |
| **Negative emotions** | **COVID-19 fear** | Believing that there is scaremongering about COVID-19. |
|  | **Public’s negative attitude towards vaccination** | Any reference to the public having a majority negative attitude towards the COVID-19 vaccines. |
|  | **Sarcasm** | A comment involving sarcasm pertaining to COVID-19 vaccines. |
|  | **Vaccine fear and anxiety** | Reference to fear and anxiety caused by COVID-19 vaccine concerns. |
| **Autonomy** | **Paternalism advertising** | Reference to the UK government promoting the COVID-19 vaccine in a paternalistic way. |
|  | **Vaccine ethics** | Reference to the ethics of the COVID-19 vaccine, e.g., considering the delay of the second Pfizer vaccine dose or approval of COVID-19 vaccines without knowing long-term side effects as unethical. |
|  | **Freedom to make decisions** | Reference to needing autonomy when making decisions about the COVID-19 vaccine, right to accept/refuse vaccines. |
|  | **Can take care of self without vaccines** | Reference to using health behaviours to protect against COVID-19, meaning vaccines would be unnecessary. |

*Table S1: The codes used in data analysis, their definitions, and initial subthemes. Colour-coding has been used to group together related subthemes.*

Appendix 2: visual map displaying how INitial subthemes were reviewed and final themes were formed


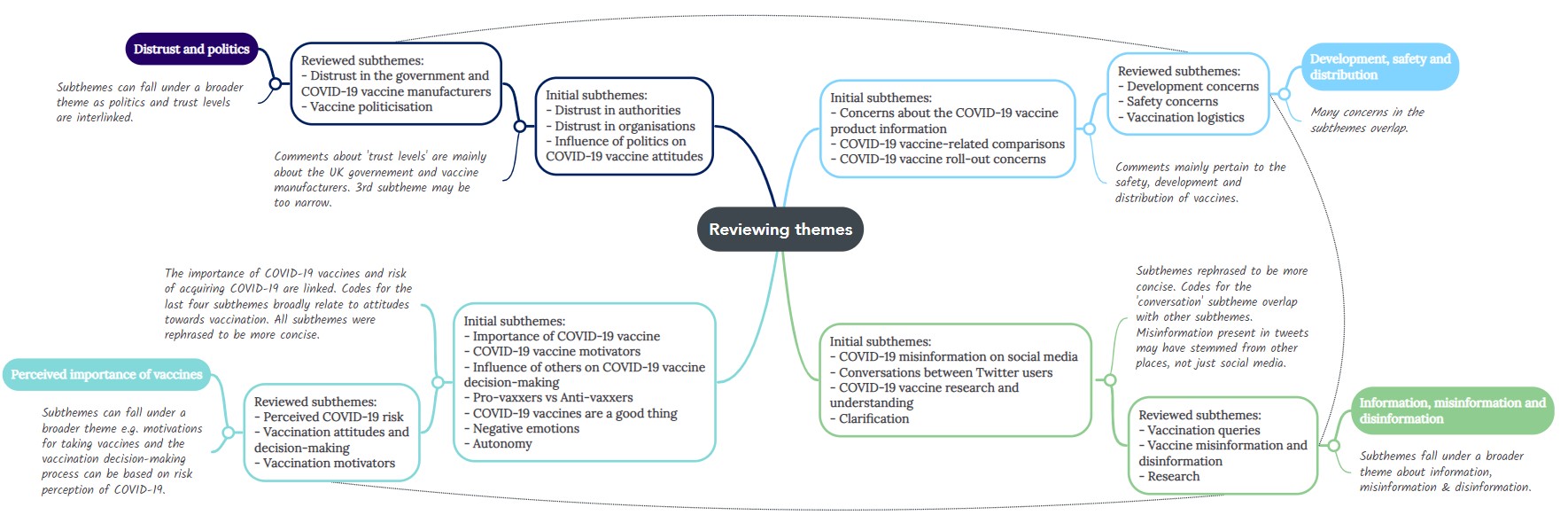


Figure S1: Visual map displaying how initial subthemes were reviewed and final themes were formed.
